# Effects of Entecavir on Hepatitis B Virus Covalently Closed Circular DNA in Hepatitis B e Antigen-Positive Patients with Hepatitis B

**DOI:** 10.1371/journal.pone.0117741

**Published:** 2015-02-03

**Authors:** Ming Shi, Wan-Li Sun, Yan-Yan Hua, Bo Han, Long Shi

**Affiliations:** Department of Laboratory Medicine, No. 6 Hospital of Dalian, Dalian, China; The University of Hong Kong, HONG KONG

## Abstract

The aim of this study was to assess the effect of 48-week entecavir therapy on serum and intrahepatic hepatitis B virus, covalently closed circular DNA (HBV cccDNA) levels in hepatitis B e antigen (HBeAg)-positive patients. A total of 120 patients with HBeAg-positive chronic hepatitis were treated with entecavir for 48 weeks. Serum HBV markers, total HBV DNA, and HBV cccDNA levels were measured at baseline and week 48. Biopsies from 20 patients were available for both intrahepatic total HBV DNA and cccDNA testing at these timepoints. HBV cccDNA levels were decreased from a median level of 5.1×10^6^ copies/mL at baseline to a median level of 2.4×10^3^ copies/mL at week 48. Reduction magnitudes of HBV cccDNA in patients with normalized alanine aminotransferase levels and those undergoing HBeAg seroconversion were significantly greater than those in alanine aminotransferase-abnormal and HBeAg positive patients. Intrahepatic HBV cccDNA was decreased significantly after 48 weeks of treatment, but could not be eradicated. In conclusion, treatment of HBeAg-positive hepatitis B patients with entecavir for 48 weeks decreased serum and intrahepatic HBV cccDNA significantly, and the magnitude of HBV cccDNA reduction was related to total HBV DNA decrease, alanine aminotransferase normalization, and HBeAg seroconversion.

## Introduction

Hepatitis B virus (HBV) infection affects more than 350 million individuals worldwide, causing acute and chronic hepatitis, liver cirrhosis, and hepatocellular carcinoma [1]. When HBV enters hepatocytes, the partially double-stranded, relaxed circular DNA (rcDNA) genome will convert into a covalently closed circular DNA (cccDNA) episome in the nucleus of infected cells [2]. The cccDNA provides the transcriptional template for viral and messenger RNAs that code for the viral structural and nonstructural proteins. A peculiarity of the HBV life cycle is that the HBV genome is either encapsulated to produce virions and be secreted into the blood, or recycled back to the nucleus to maintain a pool of cccDNA, resulting in accumulation of HBV cccDNA in hepatocyte nuclei at a level of about 5–-50 copies per cell during chronic infection [3]. Persistence of cccDNA in hepatocytes plays a key role in viral persistence, reactivation of viral replication after cessation of antiviral therapy, and resistance to therapy [4].

Several studies have shown that HBV cccDNA is detectable in the serum of patients with HBV infection [5–7], and serum HBV cccDNA levels are correlated with intrahepatic cccDNA levels [8]. This implies that measurement of serum cccDNA levels partially reflects intrahepatic cccDNA levels, which allows for serial assessment of intrahepatic cccDNA levels without the necessity of repeated liver biopsies during antiviral therapy [8].

Antiviral therapy decreases serum HBV cccDNA levels significantly. Lamivudine treatment for 12 months results in a two-log reduction in serum HBV cccDNA [9]. Combination therapy with peg-interferon and adefovir leads to a marked decrease in intrahepatic HBV cccDNA, which is significantly correlated with reduced hepatitis B surface antigen [10]. Forty-eight weeks of adefovir therapy decreases intrahepatic cccDNA by 0.8 log copies/cell [6]. However, studies in woodchuck models show that the reduction in cccDNA is caused by the loss of hepatocytes rather than a reduction in cccDNA content within these cells [11], and clinical relapse after discontinuation of antiviral therapy may be due to residual cellular HBV cccDNA [12].

Entecavir is widely used for the treatment of chronic HBV infection in China. Few studies, however, have focused on its effects on serum and intrahepatic HBV cccDNA. In the present study, we evaluated the effect of entecavir treatment on serum and intrahepatic HBV cccDNA levels in Chinese patients with hepatitis B e antigen (HBeAg)-positive chronic hepatitis B using a TaqMan real-time polymerase chain reaction (PCR) assay. This assay has a good linear range when 100 −10^7^ copies of HBV cccDNA are used as a template, while a zero background is observed using HBV viral genome DNA as a template [6].

## Patients and Methods

### Patients, treatment regimen, and serum samples

A total of 120 patients with chronic hepatitis B were treated with entecavir. All patients were over 16 years of age, positive for hepatitis B surface antigen (HBsAg) for at least 6 months and positive for HBeAg with a HBV DNA level of more than 100,000 copies per mL, and a serum alanine aminotransferase (ALT) level greater than twice the normal range. Exclusion criteria included co-infection with hepatitis A, C, D, or E viruses or HIV, decompensated liver diseases or hepatocellular carcinoma, a history of alcohol or drug abuse within 1 year prior to enrollment in the study, other possible causes of chronic liver damage, and previous treatment for chronic hepatitis B. Serum samples were taken from all patients at baseline and week 48, and stored at −70°C until used for the measurement of HBV DNA and cccDNA levels. Biopsy specimens were collected from 20 patients before and after therapy, and stored at −70°C until experimental analysis.

### Assays for ALT, HBV markers, and HBV DNA

HBsAg, hepatitis B surface antibody (HBsAb), HBeAg, hepatitis B e antibody (anti-HBe), and hepatitis B core antibody (anti-HBc) were measured using commercially available reagents for an enzyme linked immunosorbent assay (Kehua Biotech, Shanghai, China) at baseline and every 30 days thereafter. ALT activity was determined kinetically on a Beckman CX5 automatic biochemistry analyzer with a Beckman diagnostic kit (Beckman-Coulter, Brea, CA, USA). HBV DNA levels were tested using TaqMan real-time PCR reagents (Fosun Diagnostics, Shanghai, China) on ABI 7500 (Life Technologies, Foster City, CA, USA). The lower limit of the detection was 15 IU/mL according to the manufacturer’s package insert. All reagents used were approved by the State Food and Drug Administration of China for *in vitro* diagnosis use.

### Quantification of serum cccDNA

Serum HBV cccDNA levels were measured by real-time PCR as previously described, with a slight modification [6]. DNA was extracted from serum using a QIAamp DNA Mini kit (Qiagen, Shenzhen, China). Before cccDNA amplification, aliquots of each DNA sample were treated with plasmid-safe DNase (Epicentre, Madison, WI, USA). In brief, DNA extracted from 100 μL of serum was diluted to 100 μL with water. A 50-μL aliquot was heated at 75°C for 5 min and immediately placed on ice for 3–5 min. The DNA was then digested with 10 units of plasmid-safe DNase in the presence of 1× buffer and 1 mM ATP to destroy the single-strand DNA present in the rcDNA. Real-time PCR was performed on an ABI7500 (Life Technologies) using a 50-μL reaction volume containing 20 ng of DNA (for cccDNA quantification, a volume equivalent to 20 ng prior to DNase treatment was used), 2.5 mM MgCl_2_, 0.5 μM of forward and reverse primers, 0.4 μM probe. Forward and reverse primers were 5′- TCCCCGTCTGTGCCTTC-3′ (nt1549-nt1565) and 5′-CCCCAAAGCCACCCAA-3′ (nt1904-nt1889) for cccDNA amplification, respectively. The TaqMan probe was 5′-FAM-ATCTGCCGGACCGTGTGC-TAMRA-3′. The PCR cycling conditions consisted of an initial denaturing step at 95°C for 10 min, followed by 40 amplification cycles of 93°C for 15 s and 61°C for 1 min.

### Quantification of intrahepatic HBV total DNA and cccDNA

Intrahepatic HBV total DNA and cccDNA were detected using the same method as described above. DNA was extracted from 5 mg of liver biopsies using a QIAamp DNA Mini kit (Qiagen) and digested by plasmid-safe DNase (Epicentre) as described above. HBV total DNA was detected using the same commercial TaqMan real-time PCR kit (Fosun Diagnostics). Glyceraldehyde-3-phosphate dehydrogenase (GAPDH), a single-copy housekeeping gene presents in humans, was detected in the same sample and used to estimate the number of cells represented in each PCR reaction. For GAPDH amplification, the forward and reverse primers were 5′- CCAGGTGGTCTCCTCTGACTT-3′ and 5′-GTTGCTGTAGCCAAATTCGTTGT-3′, respectively. The TaqMan probe was 5′-HEX-AACAGCGACACCCACTCCTCCACC-TAMRA-3′. Serial dilutions of genomic DNA were used as standards to quantitate GAPDH DNA from liver biopsies. The performance of the assay for intrahepatic total HBV DNA was validated by the manufacturer and the lower limit of detection was 50 copies per mg of liver tissue (25 copies/10^6^ cells). The lower limit of detection of intrahepatic HBV cccDNA was 30 copies per mg of liver tissue (15 copies/10^6^ cells), according to serial dilution experiments of HBV cccDNA extracted from liver tissues. All the results were converted into copies/10^6^ cells according to the GAPDH level.

### Statistical analysis

Statistical analyses were performed with the Statistical Program for Social Sciences (SPSS 19.0 for Windows; SPSS, Chicago, IL, USA). Continuous variables with skewed distribution were tested using the Mann–Whitney test. Correlation between two variables was tested using Spearman’s correlation analysis after logarithmic transformation of the data. Statistical significance was denoted as a *P* value less than 0.05.

### Ethics

The study was conducted according to the guidelines of the Declaration of Helsinki and the principles of Good Clinical Practice. The Ethics Committee of No. 6 Dalian Hospital approved this study. All patients gave written informed consent. Two patients were aged between 16 and 18 years and signed the consent documentation themselves under the written permission of their parents, in which the parents indicated that they fully understood the contents of the informed consents and agreed to the signature of their children. As such, no informed written consent was obtained from the next of kin, caretakers, or guardians on behalf of the minors/children enrolled in this study. Verbal consent was not accepted. The Ethics Committee of No. 6 Dalian Hospital agreed on the procedure.

## Results

### Baseline characteristics of patients

The baseline characteristics of patients before treatment are summarized in Table 1. All patients were of Chinese ethnicity, HBeAg positive, and not treated previously with antiviral agents.

**Table 1:** Baseline patient characteristics.

Characteristic		Patients (n = 120)
Male sex (%)		84 (70%)
Age (yr)		
	Median	28
	Range	16–47
Weight (kg)		
	Median	60
	Range	45–72
HBV DNA (copies/ml)		
	Median	6.8 × 10^7^
	Range	1.5× 10^7^–3.2 × 10^8^
ALT (U/L)		
	Median	136
	Range	66–320.00
ALT > 2 times ULN		120 (100%)
HBeAg-positive		120 (100%)

ULN: Upper Limit of Normal.

### Serum total HBV and cccDNA reduction after treatment

Among the 120 patients receiving entecavir treatment, 79 (65.8%) and 86 (71.2%) had undetectable total HBV DNA and cccDNA, respectively, in serum. There was a significant reduction in total HBV DNA levels from a median level of 6.8×10^7^ copies/mL (range, 1.5×10^7^ to 3.2×10^8^ copies/mL) at baseline to a median level of 2.5×10^3^ copies/mL (range, undetectable to 2.2×10^6^ copies/mL) at week 48. The cccDNA levels were also decreased from a median level of 5.1×10^6^ copies/mL (range, 3.1×10^5^ to 2.6×10^7^ copies/mL) at baseline to a median level of 2.4×10^3^ copies/mL (range, undetectable to 6.7×10^4^ copies/mL) at week 48. Because most patients demonstrated undetectable serum HBV DNA and cccDNA at week 48, only patients with detectable results were included for calculation of the median. The magnitudes of total HBV DNA and cccDNA reduction were well correlated (*r* ^*2*^ = 0.79, *P*<0.01). The magnitude of reduction in serum cccDNA was lower than that of total HBV DNA, but did not reach the statistical significance [3.28 (range 1.30–4.18) vs 3.56 (range 1.32–5.63), *P*>0.05].

### Correlation of serum total HBV and cccDNA levels with ALT levels

The virological and biochemical responses of 48-week entecavir therapy are summarized in Table 2. Patients receiving entecavir showed a significant reduction in ALT levels from a median level of 136 U/L (range, 66–320 U/L) at baseline to 48 U/L (range, 26–83 U/L) at week 48. The magnitudes of serum HBV cccDNA and ALT reduction were not significantly correlated (*r* ^*2*^ = −0.022, *P*>0.05). However, the reduction magnitude of serum HBV cccDNA in ALT-normalized patients (<30 U/L) was greater than that in ALT-abnormal patients (*P*<0.01).

**Table 2:** Virological and biochemical responses following 48-week entecavir therapy.

Patients	Week 0	Week 48	
N = 120	Median (range)	Median (range)	p
HBV DNA (copies/mL)	6.8× 10^7^ (1.5× 10^7^ -3.2× 10^8^)	2.5 × 10^3^ (undetectable -2.2× 10^6^)*	< 0.01
cccDNA (copies/mL)	5.1 × 10^6^ (3.1 × 10^5^ -2.6 × 10^7^)	2.4× 10^3^ (undetectable -6.7 × 10^4^) *	< 0.01
ALT (U/L)	136 (66–320)	48 (26–83)	< 0.01

* Only detectable samples were included for calculation of the median.

### Correlation of serum total HBV and cccDNA levels with HBeAg seroconversion

At week 48, HBeAg seroconversion was observed in 62 (51.7%) patients treated with entecavir. Patients undergoing HBeAg seroconversion had significantly greater reductions of median logarithmic total HBV [4.36 (range, 2.01–6.39) versus 2.51 (range, 1.32–3.96), *P*<0.01] and cccDNA levels [3.95 (range, 1.98–5.17) versus 1.70 (range, 1.30–3.56), *P*<0.01] than those who failed to seroconvert.

### Intrahepatic total HBV DNA and cccDNA

Biopsies from 20 patients at baseline and at week 48 were available for intrahepatic total HBV DNA and cccDNA analysis. All 20 patients demonstrated undetectable HBV DNA and cccDNA in the serum at week 48, but all had detectable intrahepatic HBV DNA and cccDNA. However, the levels of intrahepatic total HBV DNA and cccDNA were decreased significantly after 48 weeks treatment with entecavir. At baseline, intrahepatic HBV cccDNA was a small part of the total HBV DNA load, but at week 48, the majority of the total HBV DNA consisted of cccDNA (Table 3). Eight (40%) of the patients showed HBeAg seroconversion at week 48.

**Table 3:** Intrahepatic HBV total DNA and cccDNA after 48-week entecavir therapy in HBeAg-positive patients.

Items	N	Baseline	Week 48	p
Intrahepatic HBV total DNA	20	3.5×10^7^	4.5×10^3^	< 0.01
(Copies/10^6^ cells, Median, range)		(4.8×10^6^~6.7×10^8^)	(7.2×10^2^~6.1×10^4^)	
Intrahepatic HBV cccDNA	20	1.3×10^6^	3.6×10^3^	< 0.01
(Copies/10^6^ cells, Median, range)		(4.5×10^4^~2.1×10^7^)	(6.2×10^2^~5.1×10^4^)	
Percentage of HBV cccDNA (%)	20	3.4 (0.2–28.4)	85.0 (43.1~96.2)	< 0.01

## Discussion

Entecavir is effective in the suppression of HBV replication, inducing HBeAg seroclearance, and reduces cirrhosis and hepatocellular carcinoma development. HBeAg clearance induced by entecavir is durable; however, clinical relapse after cessation of therapy is observed and is probably due to residual intrahepatic cccDNA.

In this study, we explored the effect of 48-week entecavir therapy on serum and intrahepatic total HBV DNA and cccDNA levels in patients with HBeAg-positive hepatitis B. Our results showed that, over a treatment period of 48 weeks, the serum total HBV DNA and cccDNA levels decreased by approximately four and three logs, respectively. The reduction in serum cccDNA levels roughly reflected a similar trend in intrahepatic cccDNA content.

In the present study, we also observed that patients showing HBeAg seroconversion had significantly greater reductions in median logarithmic total HBV and cccDNA levels than those who did not undergo HBeAg seroconversion. Although the magnitudes of cccDNA and ALT reductions were not significantly correlated, serum cccDNA reduction in ALT-normalized patients was significantly greater than that in ALT-abnormal patients. Serum HBV cccDNA may come from the destruction or lysis of hepatocytes, and implies the existence of replicative forms of HBV in hepatocytes. Currently, serum HBV DNA measurement is the only way to monitor the virologic response of the therapy in clinical practice. This method, however, cannot distinguish the cccDNA and rcDNA forms of HBV. If detectable, quantitative measurement of serum cccDNA together with HBV DNA may give insight into the decline profile of both the replicative and non-replicative forms of the virus without the necessity for a repeated liver biopsy. Our results, however, also indicated that even when serum HBV DNA and cccDNA were undetectable, HBV cccDNA remained in hepatic cells. Treatment with entecavir for 48 weeks, therefore, cannot eradicate HBV in patients with chronic HBV infection. Serial serum cccDNA measurement only provides information of the reduction magnitudes of HBV cccDNA, which may prove helpful for understanding the efficacy of antiviral therapy, but cannot be used to predict the eradication of HBV in the liver.

HBV cccDNA is detected in patient serum, HBV-infected hepatocytes, and extrahepatic infected cells such as peripheral blood lymphocytes [13–15]. It is likely that the cccDNA in serum originates from HBV-infected hepatocytes and extrahepatic cells owing to the lysis of infected cells, because the release of naked HBV nucleic acid into the circulation has been reported previously [16–18]. Reduction in serum and intrahepatic cccDNA was observed in antiviral therapy with lamivudine, adefovir, entecavir, and peg-interferon plus adefovir [9, 19, 20]. Studies on animal models and clinical trials indicate that the rates of total intracellular HBV DNA and cccDNA loss differ significantly under antiviral therapy [6]. Our results showed that the magnitude of reduction in serum cccDNA was lower than that of total HBV DNA, but did not reach statistical significance. This was probably the result of immune-mediated mechanisms that contribute to the clearance or destruction of infected cells [21, 22].

The HBeAg seroconversion rate was much higher than those reported previously. The exact reason for this discrepancy needs further study. However, many factors contribute to HBeAg seroconverion. HBV genotype, pre-C mutation, and base core promoter mutations are among these factors, which may in turn influence the observed rate of seroconversion.

Although several antiviral agents are available for treatment of chronic hepatitis B, entecavir is one of the most effective drugs recommended by the American Association for the Study of Liver Diseases and is widely used for the treatment of patients with chronic hepatitis B [23]. All patients experiencing viral relapse after the cessation of treatment exhibit significantly higher levels of cccDNA than subjects who do not relapse [24]. A 1-year course of treatment with entecavir cannot eradicate intrahepatic HBV cccDNA and the effect of long-term treatment on intrahepatic HBV cccDNA needs to be further investigated.

In conclusion, the present study demonstrated that entecavir therapy could decrease serum cccDNA levels by a magnitude of three logs in patients with HBeAg-positive chronic hepatitis B. Furthermore, a reduction in serum cccDNA levels was related to HBeAg seroconversion.

## References

[1] Lai CL, Ratziu V, Yuen MF, Poynard T (2003). Viral hepatitis B. Lancet, 362:2089–94. 14697813 10.1016/S0140-6736(03)15108-2

[2] Seeger C, Mason WS (2000). Hepatitis B virus biology. Microbiol Mol Biol Rev, 64:51–68. 10704474 10.1128/mmbr.64.1.51-68.2000 PMC98986

[3] Tuttleman JS, Pourcel C, Summers J (1986). Formation of the pool of covalently closed circular viral DNA in hepadnavirus-infected cells. Cell, 47:451–60. 3768961 10.1016/0092-8674(86)90602-1

[4] Caruntu FA, Molagic V (2005). CccDNA persistence during natural evolution of chronic VHB infection. Rom J Gastroenterol, 14:373–7. 16400354

[5] He ML, Wu J, Chen Y, Lin MC, Lau GK, et al (2002). A new and sensitive method for the quantification of HBV cccDNA by real-time PCR. Biochem Biophys Res Commun, 295:1102–7. 12135608 10.1016/s0006-291x(02)00813-6

[6] Werle-Lapostolle B, Bowden S, Locarnini S, Wursthorn K, Petersen J, et al (2004). Persistence of cccDNA during the natural history of chronic hepatitis B and decline during adefovir dipivoxil therapy. Gastroenterology, 126:1750–8. 15188170 10.1053/j.gastro.2004.03.018

[7] Chen Y, Sze J, He ML (2004). HBV cccDNA in patients’ sera as an indicator for HBV reactivation and an early signal of liver damage. World J Gastroenterol, 10:82–5. 14695774 10.3748/wjg.v10.i1.82 PMC4717084

[8] Wong DK, Yuen MF, Yuan H, Sum SS, Hui CK, et al (2004). Quantitation of covalently closed circular hepatitis B virus DNA in chronic hepatitis B patients. Hepatology, 40:727–37. 15349913 10.1002/hep.20353

[9] Yuen MF, Wong DK, Sum SS, Yuan HJ, Yuen JC, et al (2005). Effect of lamivudine therapy on the serum covalently closed-circular (ccc) DNA of chronic hepatitis B infection. Am J Gastroenterol, 100:1099–103. 15842584 10.1111/j.1572-0241.2005.41530.x

[10] Wursthorn K, Lutgehetmann M, Dandri M, Volz T, Buggisch P, et al (2006). Peginterferon alpha-2b plus adefovir induce strong cccDNA decline and HBsAg reduction in patients with chronic hepatitis B. Hepatology, 44:675–84. 16941693 10.1002/hep.21282

[11] Moraleda G, Saputelli J, Aldrich CE, Averett D, Condreay L, et al (1997). Lack of effect of antiviral therapy in nondividing hepatocyte cultures on the closed circular DNA of woodchuck hepatitis virus. J Virol, 71:9392–9. 9371599 10.1128/jvi.71.12.9392-9399.1997 PMC230243

[12] Yokosuka O, Omata M, Imazeki F, Okuda K, Summers J (1985). Changes of hepatitis B virus DNA in liver and serum caused by recombinant leukocyte interferon treatment: analysis of intrahepatic replicative hepatitis B virus DNA. Hepatology, 5:728–34. 4029888 10.1002/hep.1840050505

[13] Chen Y, Sze J, He ML (2004). HBV cccDNA in patients’ sera as an indicator for HBV reactivation and an early signal of liver damage. World J Gastroenterol, 10:82–5. 14695774 10.3748/wjg.v10.i1.82 PMC4717084

[14] Bourne EJ, Dienstag JL, Lopez VA, Sander TJ, Longlet JM, et al (2007). Quantitative analysis of HBV cccDNA from clinical specimens: correlation with clinical and virological response during antiviral therapy. J Viral Hepat, 14:55–63. 17212645 10.1111/j.1365-2893.2006.00775.x

[15] Mazet-Wagner AA, Baclet MC, Loustaud-Ratti V, Denis F, Alain S (2006). Real-time PCR quantitation of hepatitis B virus total DNA and covalently closed circular DNA in peripheral blood mononuclear cells from hepatitis B virus-infected patients. J Virol Methods, 138:70–9. 16962180 10.1016/j.jviromet.2006.07.019

[16] Su Q, Wang SF, Chang TE, Breitkreutz R, Henning H, et al (2001). Circulating hepatitis B virus nucleic acids in chronic infection: representation of differently polyadenylated viral transcripts during progression to nonreplicative stages. Clin Cancer Res, 7:2005–15. 11448918

[17] Zhang W, Hacker HJ, Tokus M, Bock T, Schroder CH (2003). Patterns of circulating hepatitis B virus serum nucleic acids during lamivudine therapy. J Med Virol, 71:24–30. 12858405 10.1002/jmv.10464

[18] Breitkreutz R, Zhang W, Lee M, Hoffmann A, Tokus M, et al (2001). Hepatitis B virus nucleic acids circulating in the blood: distinct patterns in HBs carriers with hepatocellular carcinoma. Ann N Y Acad Sci, 945:195–206. 11708479 10.1111/j.1749-6632.2001.tb03886.x

[19] Loustaud-Ratti V, Wagner A, Carrier P, Marczuk V, Chemin I, et al (2013). Distribution of total DNA and cccDNA in serum and PBMCs may reflect the HBV immune status in HBsAg+ and HBsAg- patients coinfected or not with HIV or HCV. Clin Res Hepatol Gas, 37: 373–383. doi: 10.1016/j.clinre.2012.11.002 23477988 10.1016/j.clinre.2012.11.002

[20] Wong DK, Yuen MF, Ngai VW, Fung J, Lai CL (2006). One-year entecavir or lamivudine therapy results in reduction of hepatitis B virus intrahepatic covalently closed circular DNA levels. Antivir Ther, 11:909–16. 17302253

[21] Thimme R, Wieland S, Steiger C, Ghrayeb J, Reimann KA, et al (2003). CD8(+) T cells mediate viral clearance and disease pathogenesis during acute hepatitis B virus infection. J Virol, 77:68–76. 12477811 10.1128/JVI.77.1.68-76.2003 PMC140637

[22] Zhou T, Guo JT, Nunes FA, Molnar-Kimber KL, Wilson JM, et al (2000). Combination therapy with lamivudine and adenovirus causes transient suppression of chronic woodchuck hepatitis virus infections. J Virol, 74:11754–63. 11090175 10.1128/jvi.74.24.11754-11763.2000 PMC112458

[23] Shi M, Wang RS, Zhang H, Zhu YF, Han B, et al (2006). Sequential treatment with lamivudine and interferon-alpha monotherapies in hepatitis B e antigen-negative Chinese patients and its suppression of lamivudine-resistant mutations. J Antimicrob Chemother, 58:1031–5. 16987866 10.1093/jac/dkl385

[24] Hagiwara S, Kudo M, Osaki Y, Takita M, Inoue T, et al (2013). Impact of peginterferon alpha-2b and entecavir hydrate combination therapy on persistent viral suppression in patients with chronic hepatitis B. J Med Virol, 85: 987–995. doi: 10.1002/jmv.23564 23588724 10.1002/jmv.23564

